# *Caenorhabditis elegans* as a Model for Microbiome Research

**DOI:** 10.3389/fmicb.2017.00485

**Published:** 2017-03-23

**Authors:** Fan Zhang, Maureen Berg, Katja Dierking, Marie-Anne Félix, Michael Shapira, Buck S. Samuel, Hinrich Schulenburg

**Affiliations:** ^1^Alkek Center for Metagenomics and Microbiome Research, Baylor College of MedicineHouston, TX, USA; ^2^Department of Integrative Biology, University of California, BerkeleyBerkeley, CA, USA; ^3^Zoological Institute, Christian-Albrechts University KielKiel, Germany; ^4^Centre National de la Recherche Scientifique, Institut de Biologie de l'Ecole Normale Supérieure, Institut National de la Santé et de la Recherche Médicale, ENS, PSL Research UniversityParis, France

**Keywords:** *Caenorhabditis elegans*, microbiome, microbiota, meta-analysis, *Enterobacter*, *Gluconobacter*, *Pseudomonas*, *Ochrobactrum*

## Abstract

The nematode *Caenorhabditis elegans* is used as a central model system across biological disciplines. Surprisingly, almost all research with this worm is performed in the absence of its native microbiome, possibly affecting generality of the obtained results. In fact, the *C. elegans* microbiome had been unknown until recently. This review brings together results from the first three studies on *C. elegans* microbiomes, all published in 2016. Meta-analysis of the data demonstrates a considerable conservation in the composition of the microbial communities, despite the distinct geographical sample origins, study approaches, labs involved and perturbations during worm processing. The *C. elegans* microbiome is enriched and in some cases selective for distinct phylotypes compared to corresponding substrate samples (e.g., rotting fruits, decomposing plant matter, and compost soil). The dominant bacterial groups include several *Gammaproteobacteria* (*Enterobacteriaceae, Pseudomonaceae*, and *Xanthomonodaceae*) and *Bacteroidetes (Sphingobacteriaceae, Weeksellaceae, Flavobacteriaceae)*. They are consistently joined by several rare putative keystone taxa like *Acetobacteriaceae*. The bacteria are able to enhance growth of nematode populations, as well as resistance to biotic and abiotic stressors, including high/low temperatures, osmotic stress, and pathogenic bacteria and fungi. The associated microbes thus appear to display a variety of effects beneficial for the worm. The characteristics of these effects, their relevance for *C. elegans* fitness, the presence of specific co-adaptations between microbiome members and the worm, and the molecular underpinnings of microbiome-host interactions represent promising areas of future research, for which the advantages of *C. elegans* as an experimental system should prove of particular value.

## Introduction

### The model organism *C. elegans* has been studied without its microbiome

The nematode *Caenorhabditis elegans* is one of the main model species in the life sciences, yet a surprisingly large percentage of more than 40% of the worm's gene repertoire is still without known function (Petersen et al., 2015). A likely reason is that this nematode is almost exclusively studied under highly artificial laboratory conditions, using a single isolate, the canonical strain N2, which shows substantial adaptations to the laboratory environment (Sterken et al., 2015). This strain is usually maintained in the presence of only a single bacterium, its laboratory food *Escherichia coli* strain OP50, while other microbes are routinely removed through a bleaching protocol (Stiernagle, 2006). Current studies largely ignore the natural ecology of *C. elegans*. The species shows a world-wide distribution, especially in temperate regions, where it is commonly found in rotting plant matter such as decomposing fruits (e.g., Frézal and Félix, 2015). In its natural habitat, the nematode's microbiome, here defined *sensu lato*, including a gut microbial community and possibly also microbes physically associated with the *C. elegans* surface, is likely a key determinant of life history (Petersen et al., 2015), in analogy to the fundamental role of the microbiota in the biology of all multicellular organisms examined to date (McFall-Ngai et al., 2013; e.g., Bosch and Miller, 2016). Until recently, only very few studies had explored the interactions between *C. elegans* and microbes from its environment (Grewal, 1991; Grewal and Wright, 1992; Venette and Ferris, 1998; Avery and Shtonda, 2003; Coolon et al., 2009; MacNeil et al., 2013; Montalvo-Katz et al., 2013).

The current paucity of microbiome studies in *C. elegans* is unexpected, because several characteristics make this nematode ideally suited for the experimental analysis of host-microbe interactions. First, *C. elegans* is highly amenable to genetic manipulation. Second, the presence of microorganisms can be efficiently controlled using the bleaching protocol, which is only survived by nematode eggs but no microbes, thus allowing cultivation of nematodes under axenic or monoxenic conditions (Stiernagle, 2006). Third, the nematode is transparent so that microbe colonization can be easily monitored in whole animals using simple microscopy. Fourth, several life history readouts relevant for studying *C. elegans*-microbiome interactions are well established: e.g., those related to stress resistance, life span, population growth, and fecundity. Taken together, *C. elegans* is a powerful experimental model to systematically analyze the effects of the microbiome on the host and *vice versa*. Due to these advantages, *C. elegans* has been used extensively for studying host-pathogen interactions, including mostly bacterial pathogens, but also fungi, microsporidia and viruses. This work has expanded our understanding of mechanism of innate immunity (Meisel and Kim, 2014; Cohen and Troemel, 2015; Dierking et al., 2016; Ewbank and Pujol, 2016; Kim and Ewbank, 2016). More recent work addressed the nematode's interactions with putative commensal and probiotic bacteria, such as *Comamonas, Bacillus subtilis, Lactobacillus*, and *Bifidobacterium*, yielding new insights into the mechanisms by which bacteria or their metabolites influence signaling, metabolism and life-history in the *C. elegans* host (reviewed in Clark and Hodgkin, 2014).

In 2016, three independent studies provided the first description of the microbiome of *C. elegans* and its natural environment. Taking complementary approaches (Table 1), they explored for the first time the interactions of *C. elegans* with its associated community of microbes (Berg et al., 2016a; Dirksen et al., 2016; Samuel et al., 2016). The aim of this review is to provide an overview of the understanding emerging from these three studies, and the potential of *C. elegans* to serve as an informative, experimentally accessible new model system for the dissection of host-microbiome interactions. We summarize the three studies, highlighting how they have started to define the natural microbiome, and combine them in a new meta-analysis revealing a signature of the *C. elegans* microbiome that is robust to the distinct study approaches used. We discuss the likely biological functions of the worm's microbiome and conclude by pointing to promising avenues for future research, which exploit the advantages of *C. elegans* as an experimental and genetic model system.

**Table 1:** **Overview of the first three systematic analyses of the *C. elegans* microbiome**.

	**Dirksen et al**.	**Samuel et al**.	**Berg et al**.
Study approach	Characterization of the microbiome of wild *C. elegans* isolates and the corresponding natural habitats	Characterization of the microbiome of *C. elegans* natural habitats	Characterization of the microbiome of *C. elegans* raised in soil and rotting fruit microcosms emulating habitats from which *C. elegans* has been previously isolated
*C. elegans* strains^a^	Wild isolates	N/A	N2
Substrates	Apples, compost, vector invertebrates, stems	Apples, orange, cactus fruit, snail, black bryony stems	Soil composted with different produce (harboring complex microbiota)
Sampling location	Germany, France, Portugal	France, Spain	USA (soil isolation)
Method of analysis	Deep sequencing of 16S rDNA V4 region	Deep sequencing of 16S rDNA V4 region	Deep sequencing of 16S rDNA V4 region
Main taxa identified^b^	*Proteobacteria* (*Enterobacteriaceae, Pseudomonadaceae, Xanthomonadaceae, Brucellaceae, Sphingomonadaceae*)	*Proteobacteria* (*Enterobacteriaceae, Acetobacteriaceae*), *Bacteroidetes, Firmicutes, Actinobacteria*	*Proteobacteria* (*Enterobacteriaceae, Pseudomonadaceae, Xanthomonadaceae, Burkholderiaceae, Aeromonadaceae, Alcaligenaceae, Rhizobiaceae*), *Bacteroidetes, Firmicutes*
Functional evaluation (effect of microbiome on life history traits)	Population growth on 24 individual bacterial isolates and on 14-taxa community under stress (high temperature, low/high osmolarity). Pathogen resistance.	Growth rates and induction of stress and immune reporters on 565 individual bacterial isolates from worm gut and/or substrates	N/A

a *Only C. elegans strains for which the microbiome was characterized*.

b *Non-exhaustive list of only some of the taxa*.

### The *C. elegans* natural microbiome

Two of the three *C. elegans* microbiome studies examined the natural microbial environments of wild *C. elegans* (Table 1) (Dirksen et al., 2016; Samuel et al., 2016). Using deep sequencing of the 16S rDNA V4 region bacterial content was profiled in an extensive set of natural habitats (substrates) of *C. elegans* from different sampling sites (Northern Germany, Portugal, and France)—i.e., compost, rotting apples, and other fruits, rotting stems, plus vector invertebrates used for dispersal. Characterized environmental microbial communities were composed of thousands of Operational Taxonomic Units (OTUs, representing bacterial taxonomic groups), demonstrating extensive diversity, dominated by *Proteobacteria, Bacteroidetes, Firmicutes*, and *Actinobacteria*. Of the over 250 bacterial genera that were identified in rotting apples, for example, the most abundant were *Enterobacteriaceae* and acetic acid-producing *Acetobacteriaceae*. Intriguingly, many bacterial phylotypes were consistently identified from quite disparate worm substrates (e.g., compost, snail, rotting apple and rotting orange), suggesting that these taxa are generally part of the natural environment of *C. elegans*.

Strikingly, the microbial composition of some of these habitats can predict the success of wild *C. elegans* populations living in them. Samuel et al. showed that large proliferating populations of *C. elegans* were more likely present in rotting apples with simple, *Alphaproteobacteria*-rich (*Acetobacteriaceae*) communities, while those with high levels of *Bacteroidetes* or potential pathogens tended to contain non-proliferating dauers (Samuel et al., 2016). In reconstruction experiments of two communities with about 20 species of natural bacteria, faster growth and reproduction of *C. elegans* was also observed when community composition resembled natural environments with proliferating *C. elegans* (80% *Proteobacteria, Alphaproteobacteria*-rich), rather than those containing non-proliferating dauers (40% *Proteobacteria*, enriched for *Gammaproteobacteria* and *Bacteroidetes*). Machine-learning based analyses suggest that specific microbial taxa are driving *C. elegans* population growth as well—i.e., both *Enterobacteriaceae* and *Acetobacteriaceae* are predictive of proliferating populations, while the converse was true for a Bacteroidetes (*Flavobacteriaceae*), and two *Gammaproteobacteria* families (*Xanthomonadaceae* and *Pseudomonadaceae*) (Samuel et al., 2016). As outlined below, various combinations of pairs of detrimental and beneficial bacteria from these families suggest that the impact of the *Bacteroidetes* is only observed at high abundance (>80% of the community), and that both beneficial and pathogenic bacteria can exert influence at low abundance (Samuel et al., 2016). These observations suggest that the impact of the microbiome is context dependent and involves a complex interplay between different community members.

**Figure 1:**
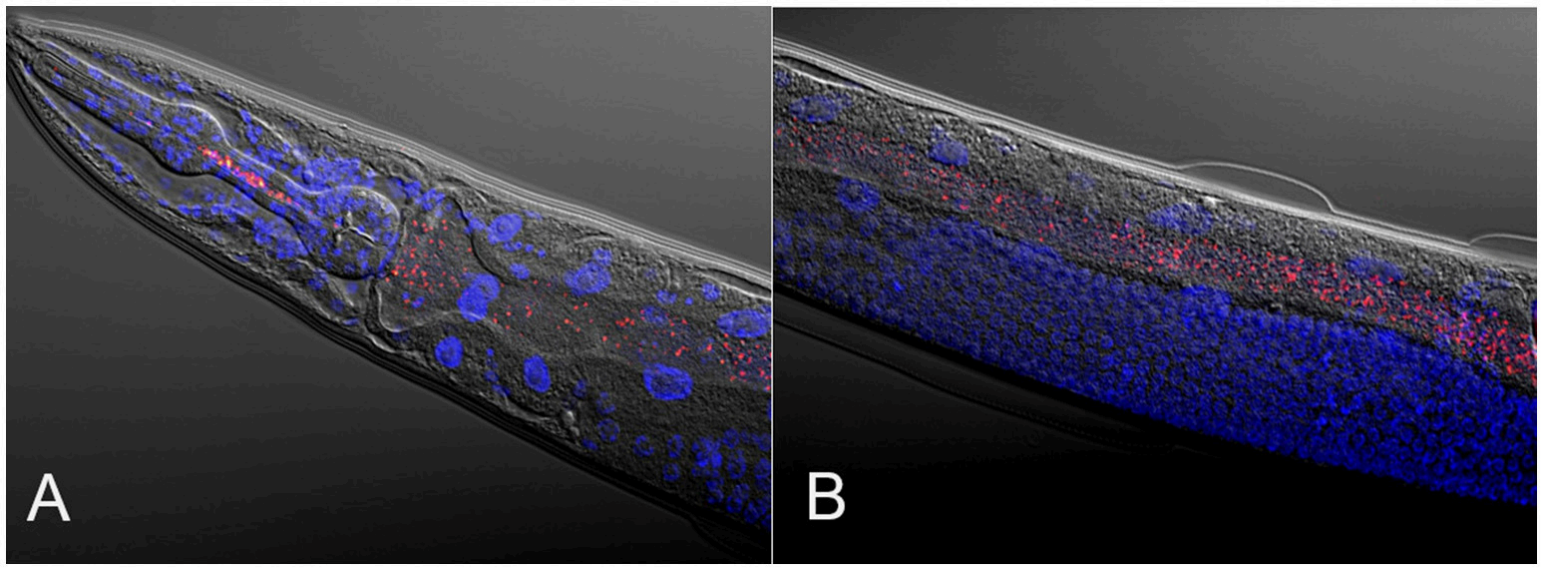
**Composite micrographs of the *C. elegans* microbiome**. **(A)** Composite micrograph of the mouth region of *C. elegans*, and **(B)** of the middle part of the worm (anterior is to the left in both cases). Nematodes were raised on an experimental microbiome based on 14 abundant bacterial taxa, followed by microscopic analysis (Dirksen et al., 2016). Bacteria are stained in red with a eubacterial FISH probe and are observed as small dots throughout the entire gut. Worm nuclei are stained in blue with DAPI. The picture in **(A)** is taken from Dirksen et al. (2016), while that in **(B)** is new, courtesy of Philipp Dirksen from the Schulenburg lab.

Dirksen and colleagues additionally analyzed the bacterial communities in natural *C. elegans* isolates (Table 1, Figure 1), in order to examine whether associated worm microbiomes differed from their corresponding substrates (Figure 1) (Dirksen et al., 2016). *Caenorhabditis elegans* from natural habitats harbored species-rich bacterial communities, including a large variety of distinct taxonomic groups (Dirksen et al., 2016). The most common OTUs were unclassified *Enterobacteriaceae* and members of the genera *Pseudomonas, Stenotrophomonas, Ochrobactrum*, and *Sphingomonas*. Moreover, the identified *C. elegans* microbiome is distinct from the microbial community of the corresponding substrates and of congeneric nematodes such as *C. remanei*, possibly suggesting the presence of a species-specific microbiome, a notion that was more recently proposed by a study examining differences in the microbiotas of different *Caenorhabditis* species (Berg et al., 2016b). Importantly, microbiomes of worms collected from different sampling sites and substrates resembled each other and, additionally, the microbial community from single worms immediately after isolation from the wild overlaps with the microbiome from worm populations expanded in the lab from over a period of several weeks (without addition of lab food) (Dirksen et al., 2016). These observations strongly suggest that *C. elegans* harbors a characteristic microbiome that is defined by its properties as a species and thus the underlying genome, irrespective of any environmental and/or geographic variations. It is yet unclear whether this characteristic microbiome is actively selected by *C. elegans* or the result of differences in nematode colonization efficacy of the various bacteria or both.

To model natural environments in the lab, work in the Shapira lab established an experimental pipeline, in which genetically-homogenous worm populations, initiated from germ-free larvae of the standard N2 strain are raised in diverse lab-based environments that emulate habitats from which *C. elegans* has been isolated in the wild (Table 1) (Berg et al., 2016a). Comparisons of microbial communities from nematodes and their corresponding microcosm environments (both analyzed by V4 16S rDNA deep sequencing) identified a characteristic *C. elegans* gut microbiome, distinct from the environment, and additionally suggested that assembly of the nematode gut microbiome was essentially a deterministic process under these conditions. The reproducibility of worm microbial communities enabled identification of a shared core microbiome, which accounted for >50% of all bacterial taxa. The analysis of nematode microbiomes from these microcosm experiments additionally revealed the presence of two distinct types, which were independent of technical variables, and differed in the abundance of core families, as well as inclusion of auxiliary taxa. Subsequent experiments evaluating the effects of temperature on microbiota composition found that changes in microbe abundance in worms were frequently in the opposite direction to changes in the environment, strongly suggesting host mediation. A more recent study confirmed that, on top of environmental-dependent variability, host genetics had a significant contribution to shaping composition of the microbiome: microbial communities were more similar in worms of the same strain than between worms of different strains and species (Berg et al., 2016b).

### Similarity and differences of the *C. elegans* microbiome across the three study approaches

**Figure 2:**
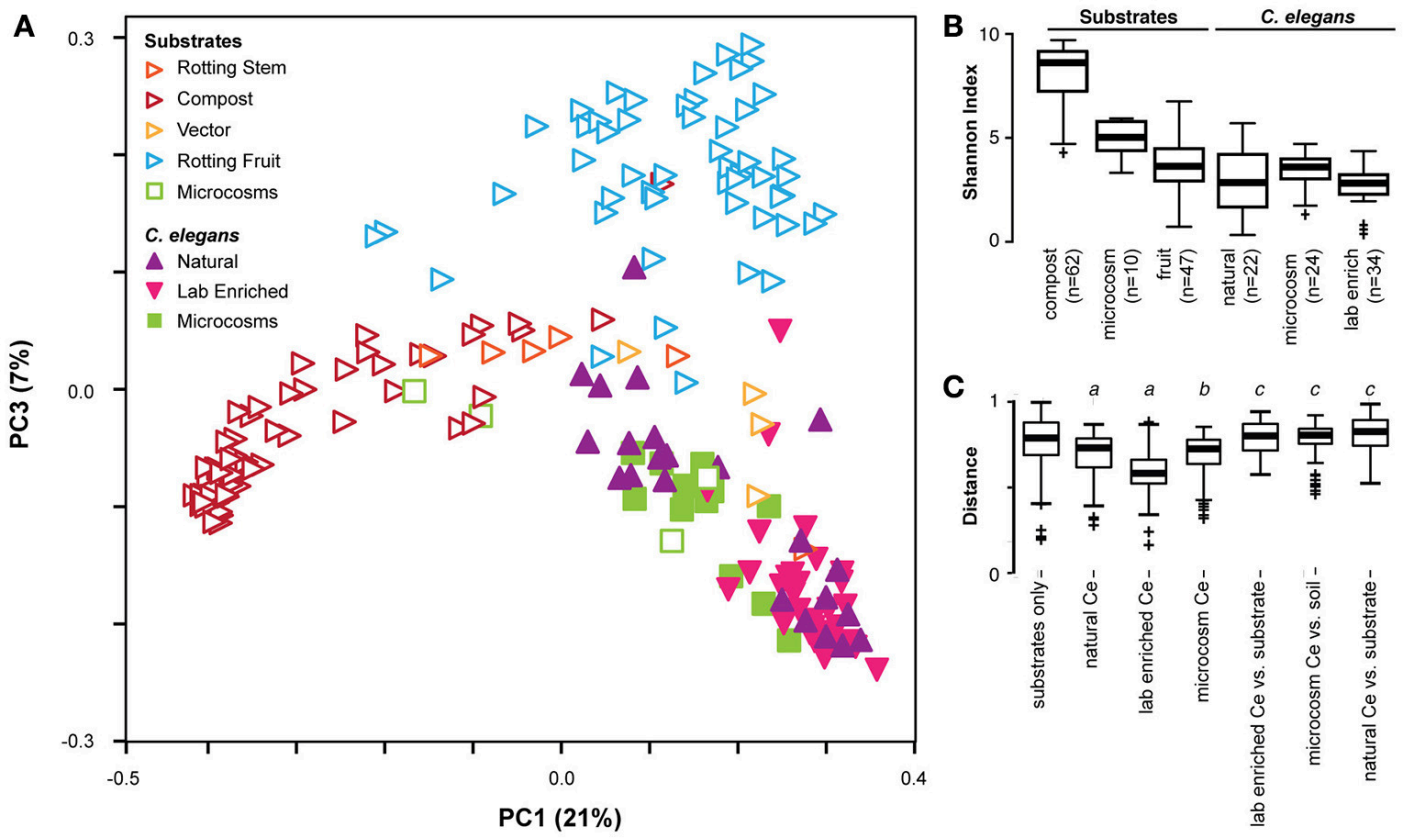
**Cross-study comparison of *C. elegans* and substrate microbiomes**. **(A)** Principle coordinate analyses based on unweighted UniFrac distances shows distinct clustering of *C. elegans* (filled) from rotting fruit or compost substrates (open) regardless of the study of origin. A three-dimensional representation of the results is provided in Supplementary Video 1. The characteristics of the included samples is presented in Supplementary Table 1, while the identified OTUs and their abundances are given in Supplementary Table 2. All microcosm data sets (given in green) are from Berg et al. (2016a). All natural and lab enriched worm data sets (given in filled purple and magenta symbols) are from Dirksen et al. (2016). The substrate data sets for rotting stem are exclusively from Samuel et al. (2016), while those for vector and rotting fruits include data from both Dirksen et al. (2016) and Samuel et al. (2016), and those for compost are exclusively from Dirksen et al. (2016). *C. elegans* microbiotas are generally less diverse than substrates as assessed by Shannon alpha diversity indices **(B)**, and exhibit more similar composition within each worm group than to substrates or between substrates **(C)**. Non-parametric *p*-values ≤0.002 are noted: a, vs. substrates; b, vs. soil microcosm; c, vs. worm group.

Bringing together the three studies enables us to better define the *C. elegans* gut microbiota by comparing microbiome compositions between worms and different substrates, as characterized by different labs with distinct study approaches and in different parts of the world (see meta-data for samples in Supplementary Table 1). Principle coordinate analysis using phylogenetic-based unweighted distances between all microbiotas, from worms and from their substrates, demonstrated that in the diversity space defined by the distribution of substrate microbiotas, worm microbiomes took up a limited sub-space (See filled symbols in Figure 2A). Analyzed worm microbiomes included (i) single worms characterized shortly after their isolation (natural worms; study by Dirksen et al., 2016), (ii) groups of worms maintained for approximately 2 weeks with their native microbiomes under laboratory conditions before microbial analysis (lab enriched worms; study by Dirksen et al., 2016), and (iii) worms of the laboratory strain N2 raised in compost microcosms (microcosm worms; study by Berg et al., 2016a). The strong overlap among these microbiomes and their distinct composition compared to the corresponding substrates strongly suggests that *C. elegans* assembles from the environment a defined, non-random microbial community, which is robust to variations in study approach (i.e., microcosms vs. natural worms), labs involved, and to perturbations due to maintenance of worms under laboratory conditions rather than their natural environments (i.e., natural vs. lab enriched worms in the study by Dirksen et al., 2016). Such a robust signature in microbiome community composition highlights the suitability of the *C. elegans* model for dissecting host-microbiome interactions and the underlying genetics irrespective of the study approach.

The presence of a distinct signature of the *C. elegans* microbiome across studies is confirmed by related statistical analyses. Unweighted distances take into consideration only presence of taxa, disregarding their abundance, and therefore represent the overall richness of microbiotas, with those in worms appearing to host a subset of the bacteria available in their environment. In agreement with this, worm microbiotas generally show substantially lower microbial diversity compared to their respective substrates, with the exception of rotting fruits that are already simple themselves (Figure 2B). They also show a greater similarity among themselves, as demonstrated by smaller inter-microbiota distances (Figure 2C). The natural *C. elegans* microbiomes exhibited the highest variation among nematode groups. Interestingly, the identified microbial communities appeared to be divided into two distinct groups. One of these clustered with almost all microbiomes from lab-enriched worms and some of the microcosm nematodes, whereas the second group clustered with a separate set of microbiomes of the microcosm nematodes (Figure 2A). Whether this division recapitulates the two microbiome types previously reported for the microcosm experiments (Berg et al., 2016a) is yet unclear. Nevertheless, the presence of distinct types among natural *C. elegans* samples suggests that nematodes may harbor different “enterotypes.” In microcosm experiments, distinct types may be attributed to environmental microbe availability and microbial competition, as suggested by ecological network analysis (Berg et al., 2016a). In wild isolates, variation in host genetics, should also be considered as a potential determinant of the presence of such two microbiome types. The importance of host genetics in this context is supported by two additional findings: The analysis of the experimental microbiome by Dirksen et al. identified a significant influence of *C. elegans* strain on bacterial community composition (Dirksen et al., 2016). A more recent set of microcosm experiments, in which different *C. elegans* strains and related species were raised on the same substrate, showed co-clustering of nematode gut microbiotas according to their genotype (Berg et al., 2016b).

**Figure 3:**
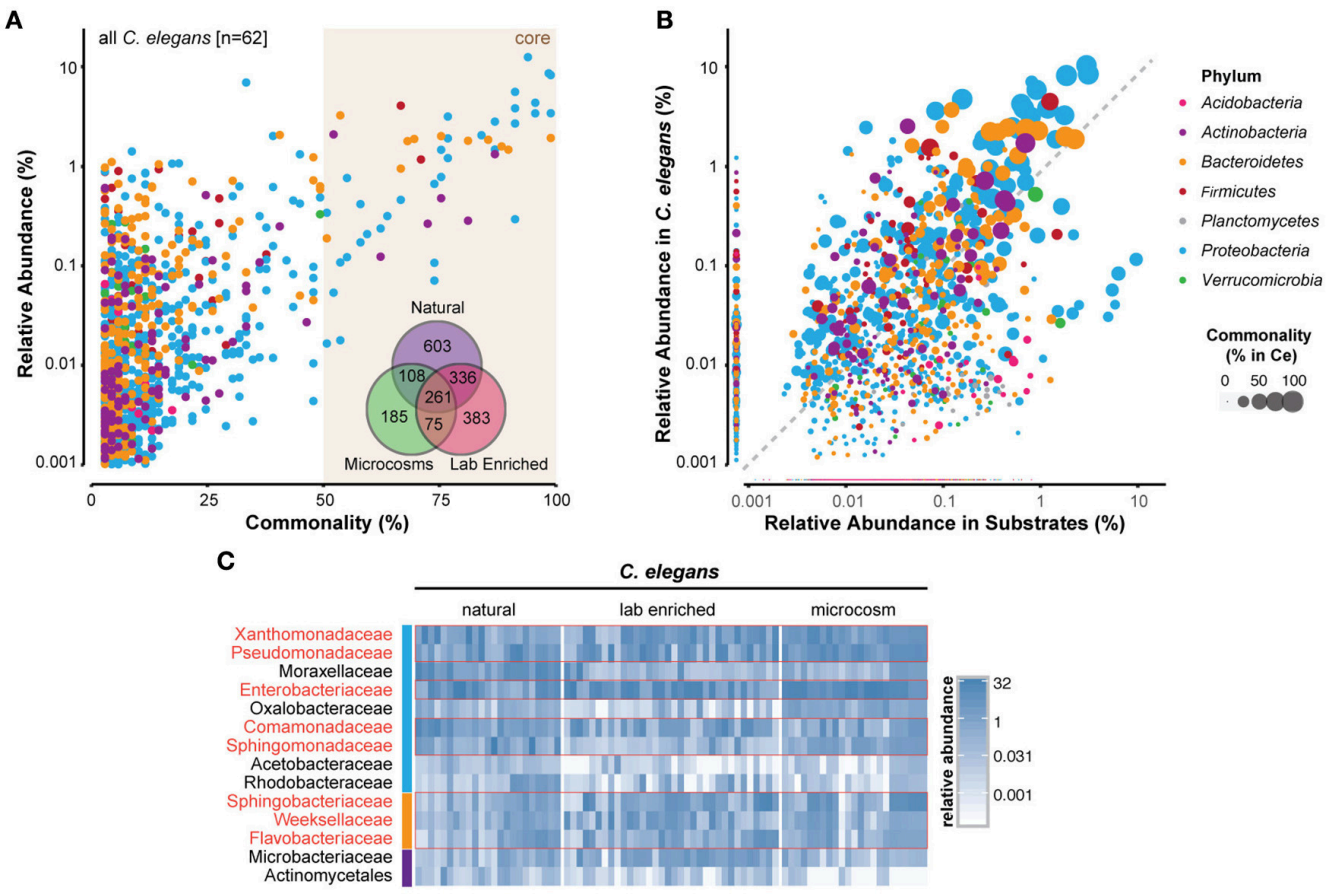
**Identification of a core microbiome of *C. elegans***. **(A)** Scatterplot of OTU-level mean relative abundance and commonality across all 62 *C. elegans* microbiomes. *Inset*, Venn diagram of the shared OTUs from each of the groups of microbiotas. **(B)** Comparison of mean relative abundance in all *C. elegans* and 119 substrate samples. The colors of circles in **(A,B)** indicate the OTUs from distinct bacterial phyla, while circle size their abundance, as highlighted in the legend on the far right. **(C)** Heatmap of 14 bacterial families that are present in 100% of the natural worm microbiomes showing abundance across samples (in %). Red boxes highlight those that are abundant also in lab-enriched and microcosm microbiotas. The colors of the vertical column on the left of the heatmap are the same as in **(A,B)** and indicate the different bacterial phyla. A more detailed heatmap, which additionally includes all substrate samples, is provided as a Supplementary Figure 1. A list of the identified OTUs and their abundances in *C. elegans* and substrates is provided as a Supplementary Table 2.

Many bacterial taxa were commonly identified among the *C. elegans* microbiotas (Figures 3A,B; Supplementary Table 2). Strikingly, 260 bacterial OTUs (operational taxonomic units) were identified in all of the studies (Figure 3A, inset; Supplementary Table 2). Several bacterial taxa were particularly abundant in worm microbiotas (Figure 3C), including three *Gammaproteobacteria*: *Enterobacteriaceae, Pseudomonadaceae*, and *Xanthomonadaceae*. Common in natural microbiotas, but less so in microcosm experiments were the *Alphaproteobacteria* members *Sphingomonadaceae*, and three *Bacteroidetes* families (*Sphingobacteriaceae, Flavobacteriaceae*, and *Weeksellaceae*) (Figure 3C). Interestingly, *Acetobacteriaceae*, which were found to correlate with large populations of proliferating *C. elegans* in rotting apples (Samuel et al., 2016), were present at low levels in all of the natural worms that were examined (Figure 3C). It is not likely that this low, yet consistent presence is due to contamination, as several other classes of bacteria present at high levels in substrates were reproducibly excluded from colonization of the worms, including for example *Planctomycetes* and most *Acidobacteria* (Figure 3B). Moreover, although *Acetobacteriaceae* are common in fruit, their abundance is much lower in compost, from which most of the characterized natural *C. elegans* were isolated. In addition to *Acetobacteriaceae*, several other *Proteobacteria* (*Moraxellaceae, Comamonadaceae*, and *Rhodobacteraceae*) and *Actinobacteria* (*Microbacteriaceae* and *Actinomycetales*) also fit into this rare, but common category within the natural *C. elegans* samples. The combination of low abundance in nematodes, yet apparent importance for their fitness, suggests that members of the *Acetobacteriaceae* and possibly also the other above listed families may serve as keystone taxa of the *C. elegans*-microbiome association with currently unknown function. Further analyses are needed to elucidate these potential roles.

The *C. elegans* core microbiota emerging from the meta-analysis is not very different from those defined by each of the separate studies. Furthermore, members of the two more prominent families, *Enterobacteriaceae* and *Pseudomonadaceae*, were isolated from *C. elegans* in earlier studies (Grewal, 1991; Ladygina et al., 2009). Together, this indicates that a significant part of the *C. elegans* microbiome is of a reproducibly defined composition that is dominated by Gram-negative bacteria, in particular fast-growing bacteria with flexible metabolisms. These bacteria are typically strong competitors, both in the environment, where they are effective colonizers of rotting fruit, and also inside the worm (Berg et al., 2016a).

### Possible functions of the worm's microbiome

Considering the consistent association between *C. elegans* and the identified bacterial taxa, it is of interest to know if and what advantages they may provide for their host. Samuel et al. demonstrated that nearly 80% of the more than 550 bacteria isolated from French substrates (BIGb and JUb collections) can individually support *C. elegans* growth (Samuel et al., 2016). The tested collections comprised 437 bacteria from rotting Orsay apples (or other habitats from sites around Paris) harboring large populations of *C. elegans* and 128 isolates from a variety of sample types and locations where *C. elegans* (and/or *C. briggsae*) animals were identified. Using a combination of physiological measures, growth rates and induction of stress and immune reporter genes, these collections of bacteria were categorized as being generally “beneficial” (promote stress-free growth), “detrimental” (impair growth, kill, activate stress/immune reporters) or “intermediate” (mixed responses). Several *Proteobacteria*, including *Enterobacteriaceae, Gluconobacter, Enterobacter, Providencia* and also most *Lactococcus* strains were more “beneficial” to *C. elegans*. More detrimental genera included Bacteroidetes, such as *Chryseobacterium* and *Sphingobacterium*, and potentially pathogenic *Gammaproteobacteria* (e.g., *Xanthomonas* and *Stenotrophomonas*). Interestingly, isolates within genera varied in influence on *C. elegans* physiology (e.g., measured with the help of stress reporter genes or growth characteristics), with the exception of *Gluconobacter*, suggesting the importance of strain-level differences in gene content (Samuel et al., 2016).

Dirksen et al. also established an experimental microbiome (Figure 1), consisting of 14 bacterial strains that were isolated from wild *C. elegans* and represented abundant genera of the worm's native microbiome (Dirksen et al., 2016). Three different *C. elegans* strains, the laboratory strain N2 and two natural isolates (all three isogenic and with different genotypes, as measured with the help of microsatellites; HS unpublished data), were grown on the experimental microbiome and bacterial populations in worms were analyzed at two different developmental stages, the fourth larval stage (L4) and adults. Analysis of the bacterial populations of these worms revealed that the developmental stage as well as the host genotype can influence the composition of the *C. elegans* microbiome. Intriguingly, certain bacteria appear to be specifically enriched in worms (when compared to the experimental microbiota on agar plates), especially *Ochrobactrum* MYb71 and *Stenotrophomonas* MYb57. This observation possibly indicates that these taxa are able to colonize the *C. elegans* intestine. At least for *Ochrobactrum* MYb71, the ability to persist in the nematode intestine was demonstrated in a separate experiment (Dirksen et al., 2016). In addition, the experimental microbiome was found to enhance worm fitness in comparison to presence of only the standard laboratory food *E. coli* OP50 and measured using population growth as proxy. Fitness was in this case increased under different stress conditions, including higher as well as lower temperatures, different media and salinities. Analysis of individual bacterial isolates further highlighted that the positive effect on fitness is likely caused by *Proteobacteria*; especially representatives of the genera *Pseudomonas, Achromobacter, Acinetobacter*, and *Comamonas* associated with substantially larger population growth than that observed under control conditions (Dirksen et al., 2016).

The best-characterized contributions of gut microbes were to host immunity. The Shapira lab previously identified a *Pseudomonas mendocina* gut isolate that conferred resistance to infection. Raising worms on the isolate protected worms from subsequent exposure to pathogenic *P. aeruginosa*, slowing-down colonization and killing (Montalvo-Katz et al., 2013). This protection was found to be provided by low-level activation (or priming) of p38 signaling, a central module in *C. elegans* immunity (Kim et al., 2002; Troemel et al., 2006; Shivers et al., 2010; Block et al., 2015). While the ability of the *Pseudomonas* commensal to provide protection from the *Pseudomonas* pathogen, may be associated with the similarity between them, other *Pseudomonas* isolates were unable to provide protection, indicating a greater specificity in recognition and immune activation. In the standard infection protocol, a prior exposure to the *P. mendocina* commensal was only able to delay colonization and death. However, in a more natural scenario, in which *P. aeruginosa* was spiked into soil with growing worms, infection could be completely averted (MB and MS unpublished data), stressing the importance of such commensals for worm fitness in its natural habitat. More recently, new isolates of *Enterobacter cloacae*, obtained either from *C. elegans* (1 isolate) or *C. briggsae* (2 isolates), were found to protect the worm from a pathogenic strain of *Enterococcus faecalis*. Interestingly, protection was specific to the host from which the bacteria were isolated: The *E. cloacae* isolate from *C. elegans* only protected its original host, but did not protect *C. briggsae*, and *vice versa* for the two *C. briggsae* isolates (Berg et al., 2016b). These findings suggest specific selection of protective symbionts by the host and possibly even some form of *Caenorhabditis-Enterobacter* co-adaptation. Such a possibility agrees with a recent demonstration, using controlled evolution experiments, of co-adaptations between *C. elegans* and a different protective bacterial strain, which reduced infection by pathogenic *Staphylococcus aureus* (Ford et al., 2016; King et al., 2016).

Two *Pseudomonas* isolates, obtained from wild *C. elegans* and distinct from *P. mendocina*, were recently shown by Dirksen et al. to inhibit the growth of six fungal strains, all similarly isolated from natural *C. elegans* (Dirksen et al., 2016). Moreover, one of these isolates protected *C. elegans* from death by a well-established fungal infection model, the ascomycete *Drechmeria coniospora* (Lebrigand et al., 2016; Zugasti et al., 2016). Fungal induced mortality was completely prevented when nematodes were exposed to the pathogenic fungus in the presence of the *Pseudomonas* isolate MYb11. It was still significantly reduced when worms were first grown on MYb11 during development and then exposed to the fungus as adults on new plates, which only contained the laboratory food *E. coli*, but not MYb11, possibly indicating a long-lasting protective effect from the latter bacterium (Dirksen et al., 2016). These studies, added to those from the Shapira lab, assign diverse anti-pathogenic contributions of Pseudomonads to *C. elegans*, which may suggest a shared history of interactions, and perhaps of evolution.

Samuel et al. expanded the spectrum of bacterial contributions to *C. elegans* pathogen resistance (Samuel et al., 2016). When *C. elegans* growth was assessed in the context of binary dilution series of three beneficial (*Gluconobacter* sp. GRb0611, *Enterobacteria* sp. JUb54, *Providencia* sp. JUb39) and three detrimental bacteria (*Serratia* sp. JUb9, *Pseudomonas* sp. GRb0427 and *Chryseobacterium* sp. JUb44), then the beneficial bacteria significantly reduced the negative effect of the detrimental taxa on worm growth. Notably, similar amounts of each equally beneficial natural bacteria (or *E. coli* OP50) did not have the same mitigating effect on each of the pathogens, suggesting that each was having its own specific protective impact rather than exhibiting a simple dilution of the concentration of a given pathogen. Whether these mechanisms occur directly on the part of the host (e.g., immune-boosting), indirectly by inhibiting growth of the pathogens, or via a related method remains to be seen.

### Future challenges

*C. elegans* possesses a microbiome with a defined signature, which can encompass a large number of bacterial taxa per individual worm. The exact presence and relative abundance of bacterial taxa can vary substantially among single *C. elegans* isolates from the wild (Dirksen et al., 2016) (Figure 2A). A particular challenge is to determine the stability of this microbial community and the strength of association of microbiota members with their host. Are bacterial strains able to persist over long time periods in nematode hosts, even if these migrate between substrates? Are such strains able to persist in dauer stages, likely used by the host for long-distance migration, and are they transmitted vertically between host generations? To what extent do *Caenorhabditis* species differ in their associated microbiomes, especially when considering host strains from different origins? Future efforts will need to catalog the specific functions of different members of the microbial community, including dominant taxa, but also the less abundant keystone taxa (i.e., those taxa consistently found at low frequency across worm samples). Do individual bacterial strains engage in mutualistic interactions with *C. elegans*—e.g., by enhancing reproductive rates of their hosts by ameliorating access to nutrition from a newly colonized substrate while the host enhances microbes' dispersal opportunities? These questions could be tested using experimental evolution approaches (e.g., Masri et al., 2015), including multi-generational propagation of *C. elegans*-microbe populations on defined substrates, and examined by microscopic analysis of bacterial colonization and persistence as well as by measuring host and bacterial fitness.

The nature of interactions between hosts and their microbiota is an important standing question that could be addressed in the *C. elegans* model. On the one hand, tight association between *C. elegans* and specific bacterial taxa may suggest co-evolution. In this case, we expect reciprocal genetic changes in *C. elegans* and individual microbial lineages, resulting in co-adaptations that are manifested in the molecular interactions among host and the specific microbes (e.g., the expression of specific microbial signaling molecules and corresponding host receptors). On the other hand, it is possible that the worm's microbiota is flexibly assembled from the environment, and consists of varying bacterial strains and taxa, which however reproducibly fulfill particular functions. However, we currently lack molecular data and also more detailed information on the functional effects of the bacteria to assess the two alternatives. Some of the available data still provides support for each of the hypotheses. That the worm microbiota is largely reproducible even when starting from diverse environments is consistent with the first possibility (Berg et al., 2016a; Dirksen et al., 2016; and meta-analysis presented here). A significant contribution of host genetics to shaping of the gut microbiota further offers support (Berg et al., 2016b; Dirksen et al., 2016). However, a strong contribution of environmental diversity to gut microbiota composition rather agrees with the second possibility (Berg et al., 2016b). That both alternatives are important is consistent with the recent model, proposed by one of us (Shapira, 2016), which suggests the gut microbiome to be divided into two parts. First, a core made of commensals with tight associations with the host, potentially sharing co-evolutionary history and possibly maintained by vertical transmission. Second, a flexible microbial pool that depends on environmental availability and can provide functional versatility, possibly advantageous in a changing environment. The two presented alternatives may actually represent opposite ends of a range of interactions. As an example for associations of a type that may lie further toward the center of this range, one can consider the acquisition of beneficial symbionts from a greater environmental diversity, relying on mechanisms permitting partner choice or checking for partner fidelity. This has been shown to occur in the colonization of the bobtail squid's light organ by *Vibrio* symbionts from the marine environment (Kremer et al., 2013; Aschtgen et al., 2016), as well as in the acquisition of *Xenorhabdus* gut-residing bacteria by the *Steinernema* entomoparasitic nematode (Murfin et al., 2015). Figuring out how *C. elegans* obtains the different members of its characteristic gut microbiota remains to be elucidated.

A particular strength of the *C. elegans* model is its amenability to genetic manipulation. This strength could be complemented by genetic analysis of individual bacterial taxa. For example, if a certain bacterial strain or mixture is found to have a strong influence on a particular phenotype, the genetics of the interaction could be dissected by forward and reverse genetic analyses, ideally in both partners. Such two-sided genetic analyses will open the possibility to characterize in detail host as well as microbial molecular processes that control host-microbiome interactions.

### Methods used for meta-analysis

The three studies applied the same 16S rRNA gene primers targeting variable region 4 (515F/806R) in bacteria (Caporaso et al., 2012). However, good quality reads were sometimes obtained with the forward primer, and sometimes with the reverse primer. In order to facilitate cross-comparisons, forward reads were used for all experiments [including re-sequenced (Illumina MiSeq) samples from Samuel et al., 2016], sacrificing in some cases the number of reads per microbiota. Additional (previously unpublished) sequences were included, with DNA isolated from *C. elegans* substrates, such as rotting apples from Orsay (FR), rotting *Petasites* stems from Ivry (FR) and slug/snail vectors from Santeuil (FR). Fastq files from the three studies were separately quality trimmed and further processed using the QIIME software package (v1.9.0) (Caporaso et al., 2010). Sequence reads with an average quality score below 25 and more than 1 ambiguous base were discarded. Sequences which passed quality filters were truncated to 150bp length to facilitate comparisons with the Illumina HiSeq reads of Berg et al. (2016a), giving rise to a dataset containing 15,197,186 reads total, with a mean of 74,862 and median of 51,932 reads per sample. Resulting fasta files were concatenated into one file, and the 16S rRNA gene sequences were further analyzed using QIIME. *De novo* OTU extraction was performed with the uclust option in QIIME. Singletons were removed from centroid consideration using the USEARCH (Edgar, 2010) suite. Resulting reads were clustered using the UPARSE algorithm at 3.0% (4 mismatches) clustering radius. Centroids were mapped to the Greengenes 13.8 database for taxonomic assignment at 97% (3.0% clustering radius) identity. Centroids failing to map to the database were evaluated with UTAX for a *de novo* taxonomic assignment. Sequences that match plant chloroplast, mitochondrial, or archaeal 16S rRNA were removed. Sequences used for our meta-analysis are available from public databases, including the European Nucleotide Archive for the Schulenburg lab data (www.ebi.ac.uk/ena; accession number ERP014530); the Sequence Read Archive database for the Samuel lab data (www.ncbi.nlm.nih.gov/sra; accession number SRS1849345), and the MG-RAST metagenomic archive for the Shapira lab data (http://metagenomics.anl.gov; accession numbers mgp13213 and mgp21372). The sample names, accession numbers, and additional meta-data are presented in Supplementary Table 1. Identified OTUs, their abundances, taxonomic classifications, and the 16S rDNA fragment consensus sequences of the most abundant *C. elegans*-associated OTUs are given in Supplementary Table 2.

Diversity indices were computed in QIIME using core_diversity_analyses.py with default parameters. For estimates of alpha-diversity (within sample), samples were rarefied to 5,000 sequences, and those samples with fewer reads were removed. Alpha diversity was determined using Shannon Index. Beta-diversity (between sample) distance matrices were generated using OTU tables rarefied to 500 observations to include as many samples possible. A phylogenetic tree of sequences representing the centroid for each OTU (a rep set tree) was generated using ClustalOmega with an enhanced version of mBed and default parameters (Sievers and Higgins, 2002). Using this rep set tree, phylogenetic-based unweighted UniFrac (Lozupone and Knight, 2005) methods were used to facilitate comparisons of presence/absence patterns (richness) between sample and substrate types. Phylogenetic relatedness of the OTUs and similarity of composition between samples are integrated to create UniFrac distance matrices that allow this comparison that were visualized by principle-coordinate analyses in QIIME. Heatmaps were generated on non-rarefied, relative abundance OTU tables and plotted in R using ggplot. Venn diagrams were created based on shared OTUs between composite (pooled) samples for each substrate or sample type. In some cases (i.e., Shannon diversity, beta diversity boxplots and heatmap), reverse read-based assessments of microcosm samples from (Berg et al., 2016a) were included to better reflect the conclusions of the original studies.

## Author contributions

MS, BS, MF, and HS conceived the work. FZ and BS generated new microbiome data. FZ, MB, MS, BS performed the meta analysis. All authors researched the literature and wrote the manuscript.

### Conflict of interest statement

The authors declare that the research was conducted in the absence of any commercial or financial relationships that could be construed as a potential conflict of interest.

## Supplementary material

Supplementary Table 1 **Overview of included data sets, sequence accession numbers, and considered meta-data**.

Supplementary Table 2 **Overview of identified operational taxonomic units (OTUs)**. Sheet I shows the identified OTUs and their abundances in the *C. elegans* samples, while sheet II those for substrate samples. Sheet III presents a list of all OTUs with their taxonomic classification. Sheet IV gives the 260 OTUs commonly found among the nematode samples, including the corresponding 16S rDNA fragment sequences.

Supplementary Video 1 **Three-dimensional visualization of the results of the Principle Coordinate Analysis**. Figure 2A of the main text shows part of the same results. Both are based on the same analysis. The color code is similar to that of Figure 2A: red, rotting stem substrates; dark red, compost substrates; orange, vector substrates; light blue, rotting fruit substrates; very light green, microcosm substrates; purple, natural worms samples; pinkt, lab enriched worms; and bright green, microcosm worm samples. All three principle coordinates are shown along the three axes.

Supplementary Figure 1 **Heatmap of the relative abundance of 14 bacterial families that are present in 100% of the natural worm microbiomes**. See legend on the right for abundance levels. Taxa and boxes in red highlight those that are abundant also in lab-enriched and microcosm microbiotas. The heatmap for the worm samples is also shown in Figure 3C of the main text, but here extended by the substrate samples.

## References

[B1] Aschtgen M.-S. Wetzel K. Goldman W. McFall-Ngai M. Ruby E. (2016). Vibrio fischeri -derived outer membrane vesicles trigger host development: OMV deliver signals in the squid/vibrio symbiosis. Cell. Microbiol. 18, 488–499. 10.1111/cmi.12525 26399913 PMC4803540

[B2] Avery L. Shtonda B. B. (2003). Food transport in the *C. elegans* pharynx. J. Exp. Biol. 206, 2441–2457. 10.1242/jeb.00433 12796460 PMC3951750

[B3] Berg M. Stenuit B. Ho J. Wang A. Parke C. Knight M.. (2016a). Assembly of the *Caenorhabditis elegans* gut microbiota from diverse soil microbial environments. ISME J. 10, 1998–2009. 10.1038/ismej.2015.253 26800234 PMC5029150

[B4] Berg M. Zhou X. Y. Shapira M. (2016b). Host-Specific functional significance of caenorhabditis gut commensals. Front. Microbiol. 7:1622. 10.3389/fmicb.2016.01622 27799924 PMC5066524

[B5] Block D. H. Twumasi-Boateng K. Kang H. S. Carlisle J. A. Hanganu A. Lai T. Y.. (2015). The developmental intestinal regulator ELT-2 controls p38-dependent immune responses in adult, *C. elegans*. PLoS Genet. 11:e1005265. 10.1371/journal.pgen.1005265 26016853 PMC4446034

[B6] Bosch T. C. G. Miller D. J. (2016). The Holobiont Imperative. Vienna: Springer.

[B7] Caporaso J. G. Kuczynski J. Stombaugh J. Bittinger K. Bushman F. D. Costello E. K.. (2010). QIIME allows analysis of high-throughput community sequencing data. Nat. Methods 7, 335–336. 10.1038/nmeth.f.303 20383131 PMC3156573

[B8] Caporaso J. G. Lauber C. L. Walters W. A. Berg-Lyons D. Huntley J. Fierer N.. (2012). Ultra-high-throughput microbial community analysis on the Illumina HiSeq and MiSeq platforms. ISME J. 6, 1621–1624. 10.1038/ismej.2012.8 22402401 PMC3400413

[B9] Clark L. C. Hodgkin J. (2014). Commensals, probiotics and pathogens in the *C. aenorhabditis* elegans model: commensals in the *C. elegans* model. Cell. Microbiol. 16, 27–38. 10.1111/cmi.12234 24168639

[B10] Cohen L. B. Troemel E. R. (2015). Microbial pathogenesis and host defense in the nematode *C. elegans*. Curr. Opin. Microbiol. 23, 94–101. 10.1016/j.mib.2014.11.009 25461579 PMC4324121

[B11] Coolon J. D. Jones K. L. Todd T. C. Carr B. C. Herman M. A. (2009). Caenorhabditis elegans genomic response to soil bacteria predicts environment-specific genetic effects on life history traits. PLoS Genet. 5:e1000503. 10.1371/journal.pgen.1000503 19503598 PMC2684633

[B12] Dierking K. Yang W. Schulenburg H. (2016). Antimicrobial effectors in the nematode *Caenorhabditis elegans* : an outgroup to the Arthropoda. Philos. Trans. R. Soc. B Biol. Sci. 371:20150299. 10.1098/rstb.2015.0299 27160601 PMC4874396

[B13] Dirksen P. Marsh S. A. Braker I. Heitland N. Wagner S. Nakad R.. (2016). The native microbiome of the nematode Caenorhabditis elegans: gateway to a new host-microbiome model. BMC Biol. 14:38. 10.1186/s12915-016-0258-1 27160191 PMC4860760

[B14] Edgar R. C. (2010). Search and clustering orders of magnitude faster than BLAST. Bioinforma. Oxf. Engl. 26, 2460–2461. 10.1093/bioinformatics/btq461 20709691

[B15] Ewbank J. J. Pujol N. (2016). Local and long-range activation of innate immunity by infection and damage in *C. elegans*. Curr. Opin. Immunol. 38, 1–7. 10.1016/j.coi.2015.09.005 26517153

[B16] Ford S. A. Kao D. Williams D. King K. C. (2016). Microbe-mediated host defence drives the evolution of reduced pathogen virulence. Nat. Commun. 7:13430. 10.1038/ncomms13430 27845328 PMC5116080

[B17] Frézal L. Félix M.-A. (2015). *C. elegans* outside the Petri dish. Elife 4:e05849. 10.7554/eLife.05849 25822066 PMC4373675

[B18] Grewal P. S. (1991). Influence of bacteria and temperature on the reproduction of *Caenorhabditis elegans* (Nematoda: Rhabditidae) infesting mushrooms (Agaricus Bispor Us). Nematologica 37, 72–82. 10.1163/187529291X00079

[B19] Grewal P. S. Wright D. J. (1992). Migration of *Caenorhabditis elegans* (Nematoda: Rhabditidae) larvae towards bacteria and the nature of the bacterial stimulus. Fund. Appl. Nematol. 15, 159–166.

[B20] Kim D. H. Ewbank J. J. (2016). Signaling in the Innate Immune Response. WormBook. 10.1895/wormbook.1.83.2 26694508 PMC6369418

[B21] Kim D. H. Feinbaum R. Alloing G. Emerson F. E. Garsin D. A. Inoue H.. (2002). A conserved p38 MAP kinase pathway in Caenorhabditis elegans innate immunity. Science 297, 623–626. 10.1126/science.1073759 12142542

[B22] King K. C. Brockhurst M. A. Vasieva O. Paterson S. Betts A. Ford S. A.. (2016). Rapid evolution of microbe-mediated protection against pathogens in a worm host. ISME J. 10, 1915–1924. 10.1038/ismej.2015.259 26978164 PMC5029159

[B23] Kremer N. Philipp E. E. R. Carpentier M.-C. Brennan C. A. Kraemer L. Altura M. A.. (2013). Initial symbiont contact orchestrates host-organ-wide transcriptional changes that prime tissue colonization. Cell Host Microbe 14, 183–194. 10.1016/j.chom.2013.07.006 23954157 PMC3928804

[B24] Ladygina N. Johansson T. Canbäck B. Tunlid A. Hedlund K. (2009). Diversity of bacteria associated with grassland soil nematodes of different feeding groups: bacteria associated with grassland soil nematodes. FEMS Microbiol. Ecol. 69, 53–61. 10.1111/j.1574-6941.2009.00687.x 19453746

[B25] Lebrigand K. He L. D. Thakur N. Arguel M.-J. Polanowska J. Henrissat B.. (2016). Comparative genomic analysis of drechmeria coniospora reveals core and specific genetic requirements for fungal endoparasitism of nematodes. PLoS Genet. 12:e1006017. 10.1371/journal.pgen.1006017 27153332 PMC4859500

[B26] Lozupone C. Knight R. (2005). UniFrac: a new phylogenetic method for comparing microbial communities. Appl. Environ. Microbiol. 71, 8228–8235. 10.1128/AEM.71.12.8228-8235.2005 16332807 PMC1317376

[B27] MacNeil L. T. Watson E. Arda H. E. Zhu L. J. Walhout A. J. (2013). Diet-induced developmental acceleration independent of TOR and insulin in *C. elegans*. Cell 153, 240–252. 10.1016/j.cell.2013.02.049 23540701 PMC3821073

[B28] Masri L. Branca A. Sheppard A. E. Papkou A. Laehnemann D. Guenther P. S.. (2015). Host–pathogen coevolution: the selective advantage of *Bacillus thuringiensis* virulence and its cry toxin genes. PLoS Biol. 13:e1002169. 10.1371/journal.pbio.1002169 26042786 PMC4456383

[B29] McFall-Ngai M. Hadfield M. G. Bosch T. C. G. Carey H. V. Domazet-Lošo T. Douglas A. E.. (2013). Animals in a bacterial world, a new imperative for the life sciences. Proc. Natl. Acad. Sci. U.S.A. 110, 3229–3236. 10.1073/pnas.1218525110 23391737 PMC3587249

[B30] Meisel J. D. Kim D. H. (2014). Behavioral avoidance of pathogenic bacteria by *Caenorhabditis elegans*. Trends Immunol. 35, 465–470. 10.1016/j.it.2014.08.008 25240986

[B31] Montalvo-Katz S. Huang H. Appel M. D. Berg M. Shapira M. (2013). Association with soil bacteria enhances p38-dependent infection resistance in *Caenorhabditis elegans*. Infect. Immun. 81, 514–520. 10.1128/IAI.00653-12 23230286 PMC3553824

[B32] Murfin K. E. Lee M.-M. Klassen J. L. McDonald B. R. Larget B. Forst S.. (2015). *Xenorhabdus bovienii* strain diversity impacts coevolution and symbiotic maintenance with *Steinernema* spp. nematode hosts. MBio 6, e00076–e00015. 10.1128/mBio.00076-15 26045536 PMC4462624

[B33] Petersen C. Dirksen P. Schulenburg H. (2015). Why we need more ecology for genetic models such as *C. elegans*. Trends Genet. 31, 120–127. 10.1016/j.tig.2014.12.001 25577479

[B34] Samuel B. S. Rowedder H. Braendle C. Félix M.-A. Ruvkun G. (2016). *Caenorhabditis elegans* responses to bacteria from its natural habitats. Proc. Natl. Acad. Sci. U.S.A. 113, E3941–E3949. 10.1073/pnas.1607183113 27317746 PMC4941482

[B35] Shapira M. (2016). Gut microbiotas and host evolution: scaling up symbiosis. Trends Ecol. Evol. 31, 539–549. 10.1016/j.tree.2016.03.006 27039196

[B36] Shivers R. P. Pagano D. J. Kooistra T. Richardson C. E. Reddy K. C. Whitney J. K.. (2010). Phosphorylation of the conserved transcription factor ATF-7 by PMK-1 p38 MAPK regulates innate immunity in *Caenorhabditis elegans*. PLoS Genet. 6:e1000892. 10.1371/journal.pgen.1000892 20369020 PMC2848548

[B37] Sievers F. Higgins D. G. (2002). Clustal Omega, in Current Protocols in Bioinformatics (John Wiley & Sons, Inc.). Available online at: http://onlinelibrary.wiley.com/doi/10.1002/0471250953.bi0313s48/abstract (Accessed January 12, 2017).

[B38] Sterken M. G. Snoek L. B. Kammenga J. E. Andersen E. C. (2015). The laboratory domestication of *Caenorhabditis elegans*. Trends Genet. 31, 224–231. 10.1016/j.tig.2015.02.009 25804345 PMC4417040

[B39] Stiernagle T. (2006). Maintenance of C. elegans. WormBook. 10.1895/wormbook.1.101.1 18050451 PMC4781397

[B40] Troemel E. R. Chu S. W. Reinke V. Lee S. S. Ausubel F. M. Kim D. H. (2006). p38 MAPK regulates expression of immune response genes and contributes to longevity in *C. elegans*. PLoS Genet. 2:e183. 10.1371/journal.pgen.0020183 17096597 PMC1635533

[B41] Venette R. C. Ferris H. (1998). Influence of bacterial type and density on population growth of bacterial-feeding nematodes. Soil Biol. Biochem. 30, 949–960. 10.1016/S0038-0717(97)00176-4

[B42] Zugasti O. Thakur N. Belougne J. Squiban B. Kurz C. L. Soulé J.. (2016). A quantitative genome-wide RNAi screen in *C. elegans* for antifungal innate immunity genes. BMC Biol. 14:35. 10.1186/s12915-016-0256-3 27129311 PMC4850687

