# Supplementary figures and images for: Caenorhabditis elegans as a Model for Microbiome Research
Source: Front Microbiol. 2017 Mar 23;8:485. doi: 10.3389/fmicb.2017.00485 (PMC5362939; doi:10.3389/fmicb.2017.00485)

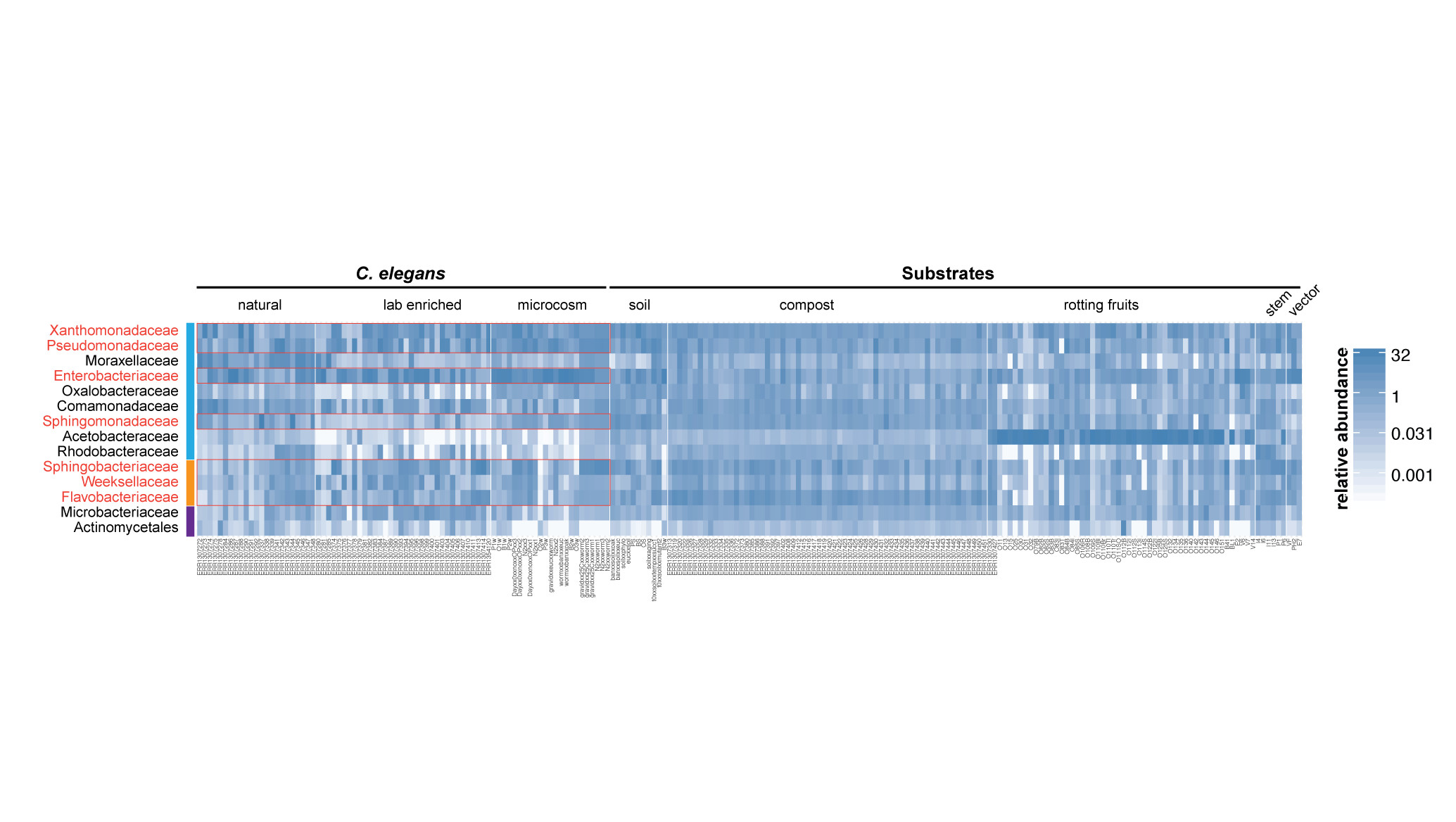

Supplement: Supplementary Figure 1 — Heatmap of the relative abundance of 14 bacterial families that are present in 100% of the natural worm microbiomes. See legend on the right for abundance levels. Taxa and boxes in red highlight those that are abundant also in lab-enriched and microcosm microbiotas. The heatmap for the worm samples is also shown in Figure 3C of the main text, but here extended by the substrate samples. [file Image1.JPEG]
